# Sensitivity of RNA viral nucleic acid-based detection of avian influenza virus, Newcastle disease virus, and African horse sickness virus on flinders technology associates card using conventional reverse-transcription polymerase chain reaction

**DOI:** 10.14202/vetworld.2022.2754-2759

**Published:** 2022-11-30

**Authors:** Khate Rattanamas, Machimaporn Taesuji, Usakorn Kulthonggate, Tippawan Jantafong, Thanongsak Mamom, Sakchai Ruenphet

**Affiliations:** 1 Master of Science Program in Animal Biotechnology, Faculty of Veterinary Medicine, Mahanakorn University of Technology, Bangkok, Thailand; 2 Clinic for Horse, Faculty of Veterinary Medicine, Mahanakorn University of Technology, Bangkok, Thailand; 3 Department of Immunology and Virology, Faculty of Veterinary Medicine, Mahanakorn University of Technology, Bangkok, Thailand; 4 Department of Pathology, Faculty of Veterinary Medicine, Mahanakorn University of Technology, Bangkok, Thailand

**Keywords:** African horse sickness virus, avian influenza virus, flinders technology associates card, Newcastle disease virus, sensitivity

## Abstract

**Background and Aim:**

The flinders technology associates (FTA) card is a cotton-based cellulose membrane impregnated with a chaotropic agent that inactivates infectious microorganisms, lyses cellular material, and fixes DNA and/or RNA within the fiber matrix. However, little is known about the effectiveness of these cards for detecting RNA viruses in animals. This study aimed to evaluate the sensitivity of RNA virus detection using conventional reverse-transcription polymerase chain reaction (RT-PCR) on FTA cards.

**Materials and Methods:**

A highly virulent Newcastle disease virus (NDV) and an avian influenza virus (AIV) with low pathogenicity were propagated using chicken embryonic eggs. Three days after inoculation, the allantoic fluid was harvested, stored at −80°C, and the stock virus was tested for virus titration. African horse sickness virus (AHSV) was obtained from a live attenuated vaccine that was dissolved and stored at −80°C. For sample preparation, each stock virus was 10-fold serially diluted and each dilution was inoculated onto an FTA card, followed by drying in a Class II safety cabinet. Both the stock virus and infected FTA card were genomically isolated using an extraction kit, FTA purification kit, and extraction kit with Tris-EDTA (TE) buffer. The target genome was then detected by one-step RT-PCR for NDV and AIV, and two-step RT-PCR for African horse sickness, including gel electrophoresis for the detection of specific nucleic acids.

**Results:**

The detection limit of stock AIV was compared on FTA cards, using the FTA purification kit, and with TE buffer with an extraction kit. The corresponding results were 1.47, 1.17, and 2.18 log_10_ EID_50_, respectively, while for NDV the results were 4.13, 4.83, and 4.84 log_10_ ELD_50_. Finally, detection limit of stock AHSV and AHSV on the FTA card extracted using TE buffer with an extraction kit were 4.30 and 4.01 log_10_ plaque-forming units, respectively.

**Conclusion:**

This study demonstrated that the detection limit or sensitivity of all tested RNA viruses on FTA cards did not differ when compared with those of the stock virus and in both methods for RNA isolation on FTA cards. These cards are suitable for collecting and transporting samples infected with RNA viruses, particularly AIV, NDV, and AHSV. Flinders technology associates cards also provide hazard-free samples, a reliable source of RNA for molecular characterization, and sufficient quantity for diagnostic applications based on nucleic acid-based detection.

## Introduction

Viral diseases in poultry, especially avian influenza (AI) and Newcastle disease (ND), are highly contagious and strongly adversely affect the profitability of the poultry industry [1–3]. Meanwhile, African horse sickness (AHS) is an infectious but noncontagious viral disease that affects all Equidae species. The causative virus exerts adverse economic effects not only through direct mortality of horses, but also through restrictions on movement introduced to reduce disease spread, the culling of infected animals, and the implementation of *Culicoides* control and horse vaccination strategies for disease control and prevention [4]. AHS was confirmed in horses in Thailand during March 2020, the causative pathogen of which was determined to belong to serotype 1 and be closely related to isolates from South Africa [5–7]. Laboratory diagnosis requires the demonstration of viral antigens or nucleic acid, whereas the characteristic clinical signs are not specific and could be confused with those of other diseases. Appropriate sample collection, sampling techniques, as well as safety measures are crucial for confirming cases of the disease while minimizing the risk of disease transmission.

The flinders technology associates (FTA) card is a cotton-based cellulose membrane impregnated with a chaotropic agent that inactivates infectious microorganisms, lyses cellular material, and fixes DNA and/or RNA within the fiber matrix [8]; thus, such samples are no longer infectious and do not pose a biohazard [9]. However, little is known about the effectiveness of FTA cards for detecting RNA viruses in animals.

This study aimed to evaluate the feasibility of FTA cards using two genomic isolation methods and to determine the detection limit of AI virus, ND virus, and AHS virus by conventional reverse-transcription polymerase chain reaction (RT-PCR), in comparison to the detection limit of stock virus.

## Materials and Methods

### Ethical approval

Ethical approval is not needed to pursue this type of study.

### Study period and location

This study was conducted from December 2021 to May 2022 at the biosecurity level-2 facilities of Virology and Molecular Diagnostic Laboratory, Faculty of Veterinary Medicine, Mahanakorn University of Technology, Bangkok, Thailand.

### Virus sample preparation

The AI virus (AIV) with low pathogenicity, namely, A/Duck/Asiancountry/2004 H9N2, and highly virulent ND virus (NDV), namely, NDV/chicken/Aseancountry/2013 [10], used in this study were provided by Prof. Thaweesak Songserm (Department of Pathology, Faculty of Veterinary Midicine, Kasetsart University). These viruses were propagated in 9-day-old chicken embryonic eggs. After allantoic fluid harvesting at 3 days postinoculation, stock viruses were aliquoted and kept at −80°C until testing. Both viruses were titrated using chicken embryonic eggs following the methods described by Ruenphet *et al*. [11].

Furthermore, the live attenuated AHS vaccine serotype 1 (Onderstepoort Biological Products, Pretoria, South Africa) was dissolved in accordance with the manufacturer’s recommendations. The dissolved vaccine virus was aliquoted for stocking and kept at −80°C until testing.

### Flinders technology associates card

Flinders technology associates card (FTA™ Classic Card; GE Healthcare, Little Chalfont, United Kingdom; lot number 17008362; expiration date July 2022) was used for this study.

### Detection limit on FTA cards

Each virus was 10-fold serially diluted using phosphate-buffered saline, inoculated on an FTA card, and kept in a Class II safety cabinet for 30 min. The infected FTA card was then punched using a 2.5- or 8.0-mm-diameter biopsy dermal puncher for AIV and NDV, respectively. The punched FTA card was subjected to RNA isolation using FTA purification reagent (Whatman™, Maidstone, Kent, United Kingdom), in accordance with the manufacturer’s recommendations, briefly placed into a microtube, supplemented with 180 μL of purification reagent, incubated at room temperature for 5 min, and then micropipetted 15 times, after which the liquid was discarded. These steps were repeated 3 times, followed by the addition of 180 μL of Tris-EDTA (TE) buffer (10 mM Tris–HCl, 0.1 mM EDTA, and pH8.0), incubation at room temperature for 5 min, pipetting 15 times, and discarding all liquid. Finally, the microtube containing the punched card was placed in an incubator at 50°C for drying for at least 15 min.

After being isolated on a punched card, AIV was amplified by one-step RT-PCR using the Superscript™ III One-Step RT-PCR system with Platinum™ Taq DNA polymerase (Invitrogen™, Life Technologies, USA). Briefly, the RT-PCR reaction contained the following: 12.5 μL of 2× reaction mix, 0.5 μL of 10 pM/μL of forward primer, 0.5 μL of 10 pM/μL of reverse primer, 1 μL of Superscript^®^ III RT/platinum^®^ taq mix, 5.5 μL of double distilled water, and the punched card after isolation. Genomic virus was amplified using a thermal cycler under the following conditions: RT at 55°C for 30 min; pre-denaturation at 95°C for 5 min; PCR for 40 cycles involving denaturation at 94°C for 1 min, annealing at 55°C for 1 min, and extension at 72°C for 1 min; and finally post-extension at 72°C for 10 min.

For an isolated card of infected NDV, Superscript™ III One-Step RT-PCR System was also used with Platinum™ Taq DNA polymerase, with the following thermal conditions: RT at 50°C for 30 min; pre-denaturation at 94°C for 15 min; PCR for 35 cycles involving denaturation at 94°C for 30 s, annealing at 55°C for 1 min, and extension at 68°C for 1 min; and finally post-extension at 68°C for 7 min.

Furthermore, in cards infected with African horse sickness virus (AHSV) including other AIV and NDV, holes of 8.0 mm diameter were punched for a second isolation method using TE buffer with a commercial extraction kit. Briefly, the punched FTA cards were mixed with 250 μL of TE buffer and incubated at room temperature for 10 min, after which the entire liquid was transferred to a new microtube for genomic extraction using an extraction kit (GF-1 viral nucleic acid extraction kit, Vivantis Technologies Sdn Bhd, Malaysia). After extraction in line with the manufacturer’s recommendations, the extracted AIV and NDV were amplified by one-step RT-PCR as described above, and AHSV was amplified by two-step RT-PCR using a Viva 2-Step RT-PCR kit with moloney murine leukemia virus (M-MuLV) RT/Taq DNA Polymerase Vivantis Technologies Sdn Bhd, Malaysia). Briefly, the RNA reverse primer mixture comprised the following: 1 μL of reverse primer mixed with 1 μL of dNTP mixture and 8 μL of extracted RNA, which was then incubated at 65°C for 5 min, after which it was immediately placed on ice for 2 min. The RT master mixture used was as follows: 2 μL of 10× buffer M-MULV, 1 μL of M-MULV reverse transcriptase, and 7 μL of double distilled water. The RNA reverse primers and the RT master mix were then combined for complementary DNA synthesis using a thermal cycler with the following thermal conditions: 42°C for 60 min, 85°C for 5 min, and 12°C for 65 min for reverse-transcription. Complementary DNA was added to the PCR master mix comprising 2 μL of 5× pulsion buffer, 0.1 μL of pulsion polymerase, 0.2 μL of 10 mM dNTP mixture, 0.5 μL of forward primer and 0.5 μL of reverse primer, and 5.7 μL of double distilled water. The mixture was then subjected to the following thermal conditions: pre-denaturation at 94°C for 5 min; PCR for 35 cycles involving denaturation at 94°C for 30 s, annealing at 53°C for 30 s, and extension at 72°C for 30 s; followed by post-extension at 72°C for 10 min. The PCR product was detected by gel electrophoresis and showed specific amplicon sizes for AIV, NDV, and AHSV of 245, 305, and 1167 bp, respectively. This study used the specific primers for AIV, NDV and AHS virus, as described in Table-1 [12–14].

**Table-1:** Primers for avian influenza virus, Newcastle disease virus, and African horse sickness virus.

Primer name	Primer sequence (5×to 3×)	Target gene	Amplicon size (bp)	Reference
FluA-M52C_F	CTTCTAACCGAGGTCGAAACG	Matrix	245	[18]
FluA-M253_R	AGGGCATTTTGGACAAAKCGTCTA			
NOH_F	TACACCTCATCCCAGACAGG	Fusion	305	[19]
NOH_R	AGTCGGAGGATGTTGGCAGC			
AHSV_F7	GTTAAAATTCGGTTAGGATG	Segment 7	1167	[20]
AHSV_R7	GTAAGTGTATTCGGTATTGA			

### Detection limit of stock virus

This study focused on the determination of the detection limit or the sensitivity of viral genomic detection on FTA cards compared with that for the stock virus. Hence, all stock viruses were also diluted and subjected to genomic extraction, RT-PCR, and gel electrophoresis as per the directions on the FTA card for determination of the detection limit.

## Results

### Virus titration

The AIV and NDV titers of the stock virus using chicken embryonic eggs were determined to be 9.17 log_10_ and 9.83 log_10_ ELD_50_/mL, respectively. However, for AHSV titration, the lowest detectable titer was 6.0 log_10_ plaque-forming units (PFU)/mL.

### Calculation of the detection limit

This study calculated the detection limit of viral samples from both stock virus and FTA cards. First, the stock virus was sampled at a volume of 200 μL at each dilution, calculated as 0.2 multiplied by the virus titer. For instance, the AIV titer of 0.2 × 9.17 log_10_ EID_50_ means that the detected titer of the undiluted stock AIV was 8.47 log_10_ EID_50_.

**Figure-1:**
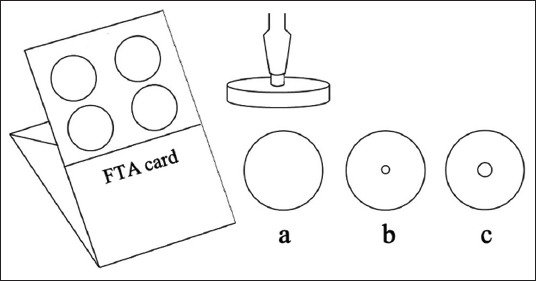
Illustration of the FTA card and biopsy dermal puncher: (a) Virus inoculation area (diameter 25 mm) = 491.07 mm^2^ (b) sampling area for 2.5 mm puncture = 4.91 mm^2^, ratio B: A = 1:100 (c) sampling area for 8.0 mm puncture = 50.28 mm^2^, ratio C: A = 1:9.76. FTA=Flinders technology associates.

Second, the ratio of virus titer on the FTA card (diameter 25 mm) to the detected titer (diameter 2.5 mm) was calculated for the detection limit at each dilution. For instance, 100 μL of undiluted AIV means that the virus titer on the FTA card was 8.17 log_10_ EID_50_/mL. Hence, the ratio of detected area to infected FTA card was 1:100 (Figure-1). Therefore, the detected titer of the undiluted virus was 6.17 log_10_ EID_50_.

Third, the ratio of 8.0 mm sampling diameter compared with infected FTA card area (diameter 25 mm) was calculated as 1:9.76 (Figure-1), so the detected titer of undiluted AIV was 7.18 log_10_ EID_50_.

### Detection limit

**Figure-2:**
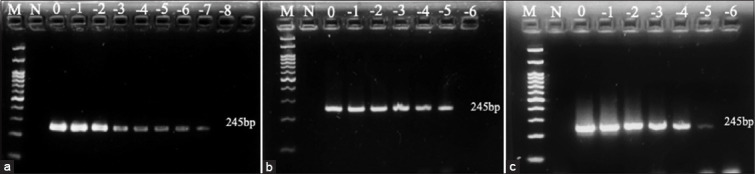
Gel electrophoresis of avian influenza virus: (a) stock virus, (b) Flinders technology associates (FTA) card using the isolation kit, (c) FTA card using TE buffer with extraction kit. M: ladder; N: negative control; 0: undiluted; −1: diluted at 1 log_10_; −2: diluted at 2 log_10_; −3: diluted at 3 log_10_; −4: diluted at 4 log_10_; −5: diluted at 5 log_10_; −6: diluted at 6 log_10_; −7: diluted at 7 log_10_; −8: diluted at 8 log_10_.

**Figure-3:**
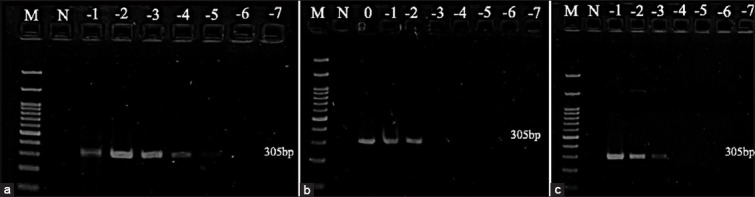
Gel electrophoresis of Newcastle disease virus: (a) stock virus (b) Flinders technology associates (FTA) card using isolation kit (c) FTA card using TE buffer with extraction kit. M: ladder; N: negative control; 0: undiluted; −1: diluted at 1log_10_; −2: diluted at 2 log_10_; −3: diluted at 3 log_10_; −4: diluted at 4 log_10_; −5: diluted at 5 log_10_; −6: diluted at 6 log_10_; −7: diluted at 7 log_10._

**Figure-4:**
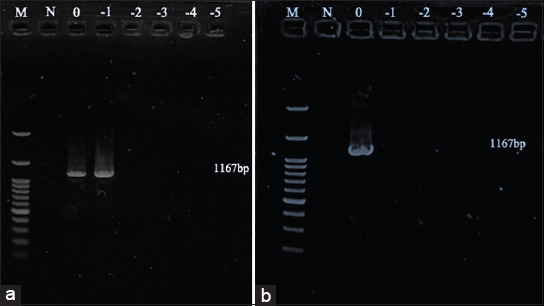
Gel electrophoresis of African horse sickness virus: (a) stock virus (b) Flinders technology associates card using TE buffer with extraction kit. M: ladder; N: negative control; 0: undiluted; 1: diluted at 1 log_10_; −2: diluted at 2 log_10_; −3: diluted at 3 log_10_; −4: diluted at 4 log_10_; −5: diluted at 5 log_10_.

The detection limits of stock virus, on an FTA card using the FTA purification kit, and on an FTA card using TE buffer with extraction kit were compared. The results showed that the detection limits of AIV were 1.47, 1.17, and 2.18 log_10_ EID_50_, respectively (Figure-2 and Table-2), while for NDV the corresponding values were 4.13, 4.83, and 4.84 log_10_ ELD_50_ (Figure-3 and Table-3). The detection limits of AHSV for stock virus and on an FTA card with TE buffer using an extraction kit were 4.30 and 4.01 log_10_ PFU, respectively (Figure-4 and Table-4).

**Table-2:** Detection limit of avian influenza virus using stock virus comparison on FTA using conventional RT-PCR.

Type of virus	Diluted and detected titer (log_10_)									Detection limit
	
	0	1	2	3	4	5	6	7	8	(log_10_ EID_50_)
Stock virus										
Detected titer	8.47	7.47	6.47	5.47	4.47	3.47	2.47	1.47	0.147	1.47
RT-PCR	+	+	+	+	+	+	+	+	-	
On FTA card by isolation kit										
Detected titer	6.17	5.17	4.17	3.17	2.17	1.17	0.17	0.017	0.0017	1.17
RT-PCR	+	+	+	+	+	+	-	-	-	
On FTA card by TE buffer										
Detected titer	7.18	6.18	5.18	4.18	3.18	2.18	1.18	0.18	0.018	2.18
RT-PCR	+	+	+	+	+	+	-	-	-	

+=Positive result by conventional RT-PCR, −=Negative result by conventional RT-PCR, RT-PCR=Reverse transcription polymerase chain reaction, FTA=Flinders technology associates

**Table-3:** Detection limit of Newcastle disease virus using stock virus comparison on FTA using conventional RT-PCR.

Type of virus	Diluted and detected titer (log_10_)									Detection limit
	
	0	1	2	3	4	5	6	7	8	(log_10_ ELD_50_)
Stock virus										
Detected titer	9.13	8.13	7.13	6.13	5.13	4.13	3.13	2.13	1.13	4.13
RT-PCR	+	+	+	+	+	+	-	-	-	
On FTA card by isolation kit										
Detected titer	6.83	5.83	4.83	3.83	2.83	1.83	0.83	0.083	0.0083	4.83
RT-PCR	+	+	+	-	-	-	-	-	-	
On FTA card by TE buffer										
Detected titer	7.84	6.84	5.84	4.84	3.84	2.84	1.84	0.84	0.084	4.84
RT-PCR	+	+	+	+	-	-	-	-	-	

+=Positive result by conventional RT-PCR, −=Negative result by conventional RT-PCR, RT-PCR=Reverse transcription polymerase chain reaction, FTA=Flinders technology associates

**Table-4:** Detection limit of African horse sickness virus using stock virus comparison on FTA using conventional RT-PCR.

Type of virus	Diluted and detected titer (log_10_)							Detection limit
	
	0	1	2	3	4	5	6	(log_10_ PFU)
Stock virus								
Detected titer	5.30	4.30	3.30	2.30	1.30	0.13	0.013	4.30
RT-PCR	+	+	-	-	-	-	-	
On FTA card by TE buffer								
Detected titer	4.01	3.01	2.01	1.01	0.1	0.01	0.001	4.01
RT-PCR	+	-	-	-	-	-	-	

+=Positive result by conventional RT-PCR, −=Negative result by conventional RT-PCR, RT-PCR=Reverse transcription polymerase chain reaction, FTA=Flinders technology associates, PFU=Plaque-forming units

## Discussion

The instability of RNA molecules is well known to impede the detection and characterization of viral RNA pathogens in the field. Several research groups, such as Cardona-Ospina *et al*. [15], Hashimoto *et al*. [16], Liang *et al*. [17], and Wannaratana *et al*. [18], have reported the benefits of using FTA cards to ensure reliable sample storage for identifying important viral diseases in animals. However, optimal handling, processing, and biosafety measures are not well established [15]. This study evaluated the detection limits of three RNA viruses (AIV, NDV, and AHSV) using conventional RT-PCR on an FTA card compared with those of the stock virus. The obtained results demonstrated that the detection limit or detection sensitivity of all RNA viruses on the FTA card did not differ from that of the stock virus. Moreover, the detection limit in this study actually reflected the real virus titer on the FTA card and could also represent the detected titer on the FTA card. For instance, on analyzing a hole with a diameter of 2.5 or 8.0 mm punched into the AIV-infected card, positive results were revealed in conventional RT-PCR at dilutions of 4 and 5 log_10_, respectively. Additional results (not included in the results table) showed that the viral titers for the two punched hole sizes in the FTA card were 2.17 and 2.18 log_10_, respectively, implying that the virus titer was uniformly distributed on the FTA card.

This study also demonstrated the results of the two methods used for genomic isolation on the FTA cards: (i) isolation using FTA purification reagent and (ii) isolation using TE buffer with a commercial extraction kit. The detection limits of AIV using the two methods were 1.17 and 2.18 log_10_ EID_50_/mL, respectively, whereas those of NDV were 4.83 and 4.84 log_10_ ELD_50_/mL. These results indicate that both methods are feasible for viral RNA isolation on FTA cards. These findings are consistent with the results of Abdelwhab *et al*. [8] and Perozo *et al*. [19], who used TE buffer with a commercial extraction kit for AIV and NDV, as well as the results of Almeida *et al*. [20], who used FTA purification reagent for *Pasteurella multocida*. However, to decide on the appropriate isolation method, some factors need to be considered, including the cost of the reagent, availability of the reagent, and the sensitivity of detection.

Interestingly, the AHSV detection limit did not differ markedly between the stock virus and the FTA card (Table-4), although the reason for this remains unknown. To the best of our knowledge, these data are the first report on the sensitivity of AHSV nucleic acid detection using an FTA card.

## Conclusion

This study demonstrated the detection limit or lowest virus titer of AIV, NDV, and AHSV on an FTA card using conventional RT-PCR and two methods for RNA isolation on FTA cards. Flinders technology associates cards are suitable for collecting and transporting samples infected with RNA viruses, particularly AIV, NDV, and AHSV. Flinders technology associates cards are not only safe by minimizing viral spread to the environment, but are also a reliable source of RNA for molecular characterization and at a sufficient quantity for diagnostic applications involving nucleic acid-based detection.

## Authors’ Contributions

KR, MT, UK, TJ, TM, and SR: Contributed to the conception of the study, designed, conducted the experiments, and analyzed the data. SR: Contributed to sample preparation. TM and SR: Drafted the manuscript. All authors have read and approved the final manuscript.
